# Measuring conflict related mortality in ten countries of the WHO Eastern Mediterranean Region (2004–2024): A scoping review

**DOI:** 10.1371/journal.pgph.0005465

**Published:** 2025-11-11

**Authors:** Esra Abdallah Abdalwahed Mahgoub, Alessandra Ferrario

**Affiliations:** Information Systems for Health Unit, Department of Science, Information and Dissemination, WHO Regional Office for Eastern Mediterranean, Cairo, Egypt; Royal Infirmary of Edinburgh, UNITED KINGDOM OF GREAT BRITAIN AND NORTHERN IRELAND

## Abstract

Armed conflict is an important contributor to the global burden of disease through direct and indirect morbidity and mortality. Yet, reliable, up-to-date, and broadly accepted estimates of the human toll are scarce. We conducted a scoping review to map methods used to estimate conflict-related deaths in ten countries of the WHO Eastern Mediterranean region using publicly available data sources. We searched three bibliomedical and scientific databases, manually searched conflict-related death databases and government websites, and reviewed websites of key humanitarian partners. In total, 69 peer-reviewed articles (from 2004 to 2024), 23 databases, 12 government sources, and 114 nutrition and mortality survey reports (from 2004 to 2024) were included. Iraq (32/69, 46%) was the most frequently studied country in the peer-reviewed literature, followed by Syria (16/69, 23%) and Yemen (14/69, 20%). About two-thirds of the studies in the peer-reviewed literature used secondary data (45/69, 65%), frequently from passive surveillance (28/69, 41%) and were national in scope (40/69, 58%). Total number of deaths was the most reported outcome in the peer-reviewed literature (63/69, 91%), followed by crude mortality rate (22/69, 32%). In 54 out of 69 studies (78%), the first author was affiliated with an institution outside the region. Among these 54 studies, only 19 had at least one co-author affiliated with an institution from the region. Databases were mostly based on passive surveillance compiling data from media reports (14/23, 61%), humanitarian organizations (9/23, 39%) and Government sources (8/23, 35%). Nutrition and mortality surveys were based on primary data collection, mostly sub-national and in some cases surveyed more frequently countries under-represented in the peer-reviewed literature. The extensive use of databases suggests a strong interest in comparable longitudinal data on mortality in conflict settings. There may be an untapped opportunity for greater use of survey data by a wider audience.

## 1. Introduction

Armed conflict is an important contributor to the global burden of disease through injury, disability, and death globally [1] and in the WHO Eastern Mediterranean Region specifically [2]. Substantial human toll results directly from the conflict (i.e., violence) and the indirectly from the lack of access to essential goods and services [1,3–5]. For example, from 2011 and 2020, an estimated 874 000 direct and indirect (excess) deaths occurred in Northwest Syria [6]. From 2017 to the end of the study in 2020, most conflict-related deaths were non-violent deaths [6].

In the past twenty years, several countries in the WHO Eastern Mediterranean Region (EMR), which comprises 22 countries and territories spanning from Northern Africa, the Middle East, all the way to Pakistan and Afghanistan, have experienced armed conflict, political instability, and waves of revolutions and military coups [7]. As of 2024, 13 out of 22 countries in the region are still embroiled in past or ongoing conflicts [8], with nine countries classified as fragile and six exhibiting very low levels of peace and stability [8].

The United Nations Sustainable Development Goal (SDG) indicator 16.1.2 tracks conflict-related deaths per 100,000 people, but it currently only includes documented deaths [9]. Accurately quantifying conflict-related deaths, especially indirect ones, is challenging due to incomplete or deteriorating registration of births and deaths as the conflict progresses; destruction of health facilities containing records and infrastructure [10]; the expertise, funding and access to affected areas required to conduct well designed mortality surveys on a regular basis and possible disincentives by the parties involved to reveal the true death toll [11,12] and often siloed efforts by the global development community [13]. As a result, reliable, up-to-date and broadly accepted estimates of the human toll are scarce. Curated media reports and passive surveillance have tried to fill the gaps but it has been shown that they are incomplete and affected by reporting bias [14,15].

NGOs and humanitarian partners have conducted most of the nutrition and mortality surveys in humanitarian settings. These are often published on their websites but not always. In this scoping review, we searched readily accessible online resources such as published peer-reviewed literature, online databases, Government websites, and websites of main development partners and humanitarian actors for studies and data on conflict-related deaths in ten countries in the EMR. We wanted to know which were the most studied countries, the methods used to estimate mortality, the outcomes reported, the country affiliation of the authors and who funded the studies and reports.

Reviewing readily available data sources is important as these are the ones most people will be able to access and therefore representing the data and information most researchers and the media will have access to and thus potentially most likely to impact public discourse.

## 2. Methods

### 2.1 Scoping review

We conducted a scoping review [16] to map study characteristics, methods used to estimate conflict-related deaths and reported outcomes in selected countries of the World Health Organization (WHO) Eastern Mediterranean Region. This scoping review adhered to the Preferred Reporting Items for Systematic Reviews and Meta-Analyses extension for Scoping Reviews (PRISMA-ScR) Checklist [16] guidelines (S1 Checklist). Although not registered, the review protocol is available upon request from the corresponding author. The study was conducted between April and September 2024.

To ensure consistency, this study defines “conflict” based on the categories outlined in the Technical Guidance Note on SDG Indicator 16.1.2 published by the United Nations Human Rights Office (OHCHR) [9]. Terrorism in this study refers only to attacks occurring during conflict.

### 2.2 Study population

Our study covered ten countries of WHO EMR considered to be in emergency by the WHO Health Emergency Programme for most of the time between the 1^st^ of January, 2004 and the 1^st^ of June 2024 and had a history of conflict as defined in Technical Guidance Note on SDG Indicator 16.1.2 [9]. These countries are Afghanistan, Iraq, Lebanon, Libya, the occupied Palestinian territory, Pakistan, Somalia, Sudan, Syrian Arab Republic, and Yemen.

### 2.3 Data sources and search strategies

We used publicly available information online. This involved electronic and manual searches of the peer-reviewed literature, databases reporting on conflict-related deaths, government websites and nutrition and mortality reports surveys in the ten study countries.

For the peer-reviewed literature, we searched three electronic databases: PubMed, Web of Science, and Global Index Medicus using the JBI three-step search strategy [17]. The search focused on three concepts: deaths, conflict, and EMR countries. We included articles published between January 2004 to the time of the search in 2024. The details of three-step search strategy and the final search strategy for each database are provided in Box A and Table A in S1 Text. The searches were conducted on July 31st and August 1st. There were no language restrictions.

We included studies on deaths caused directly by conflict, studies on indirect deaths, caused by the lack of access to basic services (health care, food, water, hygiene and sanitation), and studies on deaths by any other causes at the time of conflict. We considered studies that used conflict-related deaths as an independent variable if the aim of the study was to estimate overall mortality rates or forecast future conflict fatalities. We excluded studies that merely presented previously reported data without additional analysis, studies using conflict-related deaths from databases as an independent variable which did not aim to estimate mortality, studies focusing solely on foreign combatants, opinion articles, news articles, editorials, commentaries, replies, retracted articles, systematic reviews, scoping reviews, meta-analyses (although we scanned the reference lists of identified reviews for potentially relevant research articles), and facility-based studies unless they covered an entire country or specific catchment areas.

We used Google as part of our manual search to identify conflict related death databases and the governmental sources reporting mortality in each country using Arabic and English keywords. We reviewed conflict related death databases that primarily focus on reporting fatalities from conflicts, although they may also track other war-related consequences. We included open-source databases covering at least one of the targeted countries. Databases relying on data from other conflict-related death databases were excluded, along with subscription-based and non-functional websites, in addition to databases that only included fatalities of one professional group (e.g., humanitarian actors). We reviewed national Civil Registration and Vital Statistics (CRVS) websites to identify which Ministry within each country is responsible for recording deaths. We included the governmental sources that have an online website.

For nutrition and mortality surveys, we conducted searches on the websites of WHO EMRO, UNICEF, MSF, Action Against Hunger, ReliefWeb, OCHA, and Save the Children. The search was limited to the study’s targeted duration, employing keywords such as “mortality survey,” “nutritional survey,” and “SMART survey,” and by reviewing publications related to specific countries. We included reports that focused on mortality data as one of the survey objectives and reported mortality results. We excluded reports that analyzed a group of surveys conducted during a specified period and for which we could not retrieve the individual survey reports as we could not extract the variables of interest.

### 2.4 Data charting process

The retrieved articles were exported to Covidence [18], a software to manage and streamline the literature review process, where duplicates were removed. Two reviewers conducted the initial screening of titles and abstracts, followed by a full-text review, using pre-specified inclusion and exclusion criteria to eliminate irrelevant studies. Any discrepancies were resolved through discussion between the two reviewers to achieve consensus. Survey reports were also exported to Covidence separately and reviewed by one reviewer who also conducted the data extraction.

We developed a data extraction form to extract the variables of interest. The form was divided into three main sections: study characteristics, methods, and reported outcomes (S2 Text).

To ensure the accuracy of the form, two studies were independently extracted by three researchers. The extraction outputs were then compared and discussed to obtain feedback, leading to necessary modifications of the form. The extraction process was carried out by one reviewer with biweekly meetings to monitor progress and discuss any challenge encountered. Following Arksey and O’Malley framework [19], we did not appraise the quality of included studies.

### 2.5 Synthesis of results

The extracted data were exported from Covidence as an Excel workbook. Subsequently, data was cleaned and analyzed using Statistical Package for the Social Sciences (SPSS) version 26. Open-ended questions regarding data verification methods and challenges in obtaining data were categorized to generate quantitative results. We used the mortality typology of conflict mortality proposed by Ratnayake *et al*. (2008) [20] which characterizes crude mortality as composed of baseline and excess mortality and further subdivides excess mortality into direct and indirect deaths. These results are presented in the form of frequencies, percentages, and tables, figures, and narrative.

## 3. Results

### 3.1 Peer-reviewed literature

**Fig 1:**
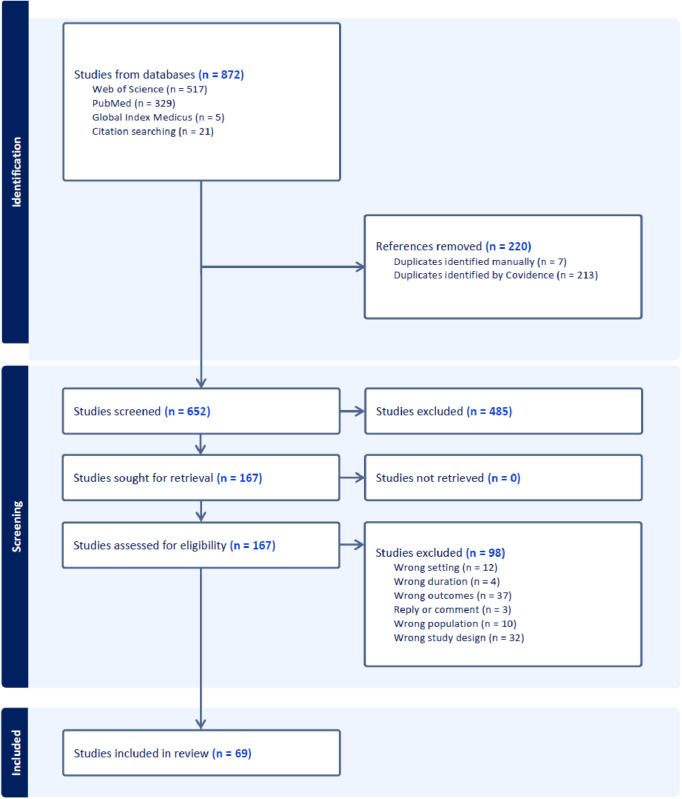
Scoping review PRISMA flow diagram.

Our search strategy for peer reviewed articles yielded 872 results in total. After removing duplicates, a total of 652 studies were screened for their titles and abstracts. Of these, 167 were examined in full text. Ninety-eight studies were excluded for not meeting the inclusion criteria. This resulted in a total of 69 studies included in the review. Fig 1 illustrates the PRISMA flow diagram.

#### 3.1.1 Study characteristics.

**Fig 2:**
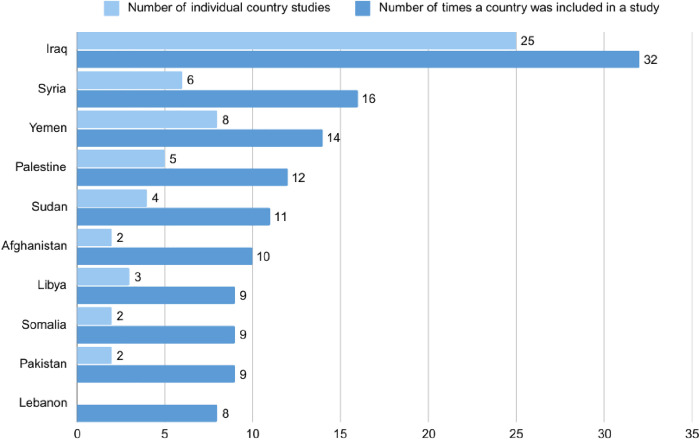
Number of peer-reviewed studies per country.

All 69 studies included were published in English. Of these, 57 focused on a single country, while 12 covered multiple countries. Most publications focused on Iraq (32/69, 46%), followed by Syria (16/69, 23%) and Yemen (14/69,20%). There were no publications focusing on Lebanon individually but the country was included in 8 of the multi-country studies (8/69, 12%) (Fig 2).

More than sixty percent of the studies were published between 2016 and 2024 (43/69, 62%). In most of the studies (54/69, 78%), the first author was affiliated with an institution outside the EMR. Of these 54 studies, only 19 had at least one co-author affiliated with an EMR institution (Table 1). Among the 15 first authors affiliated with an EMR institution, 5 were from Iraqi and 3 from Libyan institutions. Of the 54 first authors from outside the EMR, 27 were affiliated with institutions in the United States and 10 with institutions in the United Kingdom. Table A in S3 Text provides further details about the affiliations of the first authors by country and institution.

**Table 1:** Characteristics of the studies included in the peer-reviewed literature (N = 69).

			n	%
**Publication year**		2004 - 2006	5	7.2
		2007 - 2009	3	4.3
		2010 - 2012	10	14.5
		2013 - 2015	8	11.6
		2016 - 2018	16	23.2
		2019 - 2021	16	23.2
		2022 - 2024	11	15.9
**Author’s affiliation**	First author affiliation	First author affiliated with an EMR institution	15	21.7
		First author affiliated with an institution outside EMR	54	78.3
	Affiliation of other authors when the first author is affiliated with an institution outside EMR (N = 54)	None of the authors affiliated with an EMR institution	35	64.8
		At least one of the other authors affiliated with an EMR institution	19	35.2

Nearly half of the studies did not report financial support for the study (38/69, 55%). Among studies reporting a source of funding (31/69, 45%), the most common funders were the United Kingdom Foreign Commonwealth and Development Office (FCDO) (5 studies) and Surgeons OverSeas (SOS) (5 studies), followed by the Bill & Melinda Gates Foundation (4 studies) and Médecins Sans Frontières (MSF) (4 studies) (Table B in S3 Text). The studies were published in 40 different journals, the most common were BMC Conflict and Health (8 articles), The Lancet (7 articles), Prehospital and Disaster Medicine (6 articles), and PLOS Medicine (5 articles) (Table C in S3 Text).

#### 3.1.2 Methods used.

Approximately half of the studies (31/69, 44.9%) in the peer-reviewed literature examined both direct and indirect conflict-related deaths, while only 12 (17%) focused solely on indirect deaths. All studies but one (68/69, 99%) were retrospective.

Secondary data was used in most studies (45/69, 65%), while primary data was collected in 21 studies (30%), and the remaining three studies used a combination of primary and secondary mortality data. Conflict related deaths databases was the most common source of data (41%, n = 28), followed by primary data collection (24/69, 35%), Table 2.

**Table 2:** The methods used in the studies in the peer-reviewed literature (N = 69).

Methods		n	%
**Scope of the studies**	Direct conflict related deaths	26	38
	Indirect conflict related deaths	12	17
	Both direct and indirect conflict related deaths	31	45
**Study design**	Retrospective	68	99
	Prospective	1	1
**Type of mortality data**	Primary	21	30
	Secondary	45	65
	Primary and secondary	3	4
**Source of data**	Governmental reports or websites	18	26
	Humanitarian and research organizations	17	25
	Conflict related deaths databases	28	41
	Primary data collection	24	35
	Others*	8	12
**Civilian-combatant distinction**	Civilian only	30	44
	Combatants only	–	–
	Both civilians and combatants	13	19
	Not specified	26	38
**Targeted population in the studies**	General population	51	74
	Infectious diseases cases	6	9
	Women and/or children and/or adolescents	5	7
	IDPs	4	6
	Adults	2	3
	Media workers	1	1
**Geographic scope**	National	40	58
	Sub-national	29	42
**Sampling methods used by the primary data collection studies (n = 24)**	Cluster sampling	13	54
	Purposive sampling	7	29
	Respondent-driven sampling	1	4
	Simple random sampling	1	4
	Stratified sampling	2	8
**Did the study clearly specify the inclusion and exclusion criteria?**	Yes	37	54
	No	32	46

*Others: Data from previous studies [6], Grey literature review [1], Media [3]. Note that one study drew information from all three sources. Note: percentages were rounded to the nearest full digit, as result percentages may not always perfectly add up to 100%.

In Iraq, the use of household surveys to collect primary data was prominent (12 studies), followed by the Iraq Body Count database. The studies from Yemen evenly employed governmental sources and primary data collection. Among the latter, two used satellite imagery to estimate deaths, and one used web-based mortality surveys. Organizational sources were a common source, particularly in Syria, Somalia, Sudan, and Afghanistan. Notably, no governmental sources were used in Syria, Sudan, or Pakistan, and no study was found using primary data in the occupied Palestinian territory, Somalia, and Pakistan. Table D in S3 Text present the sources of data used in country specific studies per country.

Thirty studies (44%) focused solely on civilians, while 13 (19%) examined both civilians and combatants, the remainder did not specify which of these two groups were included. Most studies (51/69, 74%) investigated the general population, with 4 focusing on internally displaced persons (IDPs) and 5 concentrating on women and/or children and/or adolescents. Forty studies (58%) covered the entire country, while 29 (42%) focused on specific regions. Approximately half of the studies (32/69, 46%) did not explicitly state their inclusion and exclusion criteria. Cluster sampling was the most used method for primary data collection (13 out of 24), followed by purposive sampling in 7 studies (out of 24). None of the studies provided definitions for conflict-related deaths, direct conflict-related deaths, and indirect conflict-related deaths.

Additional details regarding the methodologies employed are presented in Table 2. Table E in S3 Text illustrates the study scopes and population per countries. The periods covered by each study per country are illustrated in Fig A to J in S3 Text.

Only twelve studies reported methods of data verification. Four studies used death certificates to verify deaths. Three studies had the data collection team supervisor or another investigator re-interview a random sample of households to cross-check the collected data. Two studies-maintained contact with primary sources to consult for missing data or verification. Two studies discussed and approved the final results with key informants. One study included cases based on multiple database documents.

#### 3.1.3 Reported outcomes.

Following the typology of mortality by Ratnayake *et al.* [20], 65% (28/43) of the studies quantifying indirect deaths, reported these deaths as part of a broader metric reflecting baseline and indirect deaths combined. Similarly, 26% (11/43) reported baseline and excess deaths combined, which also includes indirect deaths, and 26% (11/43) reported excess deaths, which include direct and indirect death. Additionally, 18.6% (8/43) reported crude death rates, which consists of both direct and indirect deaths in addition to the baseline death. Notably, only 7% (3/43) of the studies reported indirect deaths as a separate, distinct metric.

The most frequently reported outcomes were total number deaths (63/69, 91%), overall mortality rates (33/69, 48%), and age-specific mortality rates (17/69, 25%). Among studies reporting proportionate mortality, 11 reported violence-related deaths, 3 reported war-related deaths. Of those reporting age-specific mortality, 8 examined under-five mortality, and 4 neonatal mortality rates. For cause-specific mortality, 5 reported violent-specific mortality, 3 maternal mortality. Case fatality rate was reported in 6 studies. Regarding morbidity and disease burden, 19 studies reported injury rates or numbers, 6 disability rates or numbers (Table 3).

**Table 3:** The reported outcomes of the studies in the peer-reviewed literature (N = 69).

Reported outcomes categories	n	%	The specifically reported outcome	n	%
Total number of deaths	63	91			
**Overall mortality rates**	33	48	Crude mortality rate	22	67
			Proportion of deaths in the study sample ^**b**^	11	33
**Excess mortality rate/number**	11	16	Number of excess deaths	5	45
			Number and rate of excess deaths ^**b**^	5	45
			Rate of excess deaths ^**b**^	1	9
**Proportionate mortality rates** ^**b**^	20	29	Violence-related mortality rates	11	55
			War-related mortality rates	3	15
			Traffic-related mortality rates	3	15
			Injury-related mortality rates	6	30
			Non-communicable diseases related mortality rates	3	15
			Communicable diseases related mortality rates	3	15
			Non-violence related mortality rates	3	15
			Indirect-related mortality rates	1	5
			Ethnicity-related mortality rates	1	5
**Age specific mortality rates**	17	25	Under five mortality rate	8	47
			Neonatal mortality rate	4	24
			Infant mortality rate	3	18
			School-aged children and adolescents’ mortality rate	2	12
			Age-standardised mortality rate	2	12
			Adult mortality rate	1	6
			Childhood mortality rate	1	6
**Cause specific mortality rates** ^**b**^	16	23	Violent specific mortality	5	31
			Maternal mortality	3	19
			Conflict specific mortality	2	13
			Explosives injury specific mortality	1	6
			Firearm specific mortality	1	6
			Intentional Injury specific mortality	1	6
			Non-trauma specific mortality	1	6
			Assassination rate	1	6
			Other	1	6
**Morbidity and burden of disease**	22	32	Injuries (number/rate)	19	86
			Disability (number/rate)	6	27
			Years of Life Lost due to Premature Mortality (YLLs)	3	14
			Years of Healthy Life Lost due to Disability (YLDs)	3	14
			Disability-adjusted life year (DALY)	3	14
			Mean number of days of normal activity lost	1	5
**Other**	22	32	Case fatality rate	6	27
			Burial rate per day	1	5
			Ratio of civilian to media worker deaths	1	5
			Daily burial rates and number/rate of excess burials	1	5
			Crude incidence rate ratio	1	5
			Expected number of deaths	1	5
			Injured-to-killed ratio	1	5
			Likelihood of being killed	1	5
			Mean and median fatalities/day	1	5
			Percentage of change in deaths per year	1	5
			Proportion of explosion-related deaths among all injury deaths	1	5
			Ration of firearm-related fatalities to fatal explosives-related injuries	1	5
			Relative risk of death	2	9
			Unique entity estimation	1	5

a Note: percentages were rounded to the nearest full digit, as result percentages may not always perfectly add up to 100%.

b Proportion of deaths in the study sample: The percentage of individuals who died out of the total number of individuals included in the study. **Excess death rate**: The increase in the mortality rate observed during a crisis period, compared to the counterfactual mortality rate that would have occurred in the absence of the crisis. Typically expressed per unit of person-time. **Cause-specific mortality rate**: The mortality rate due to a specific cause (e.g., conflict, violence, injury, maternal causes, or deaths in detention), expressed in relation to the total population and often per unit of person-time. **Proportionate mortality**: The proportion of all recorded deaths that are attributable to a specific cause.

**Fig 3:**
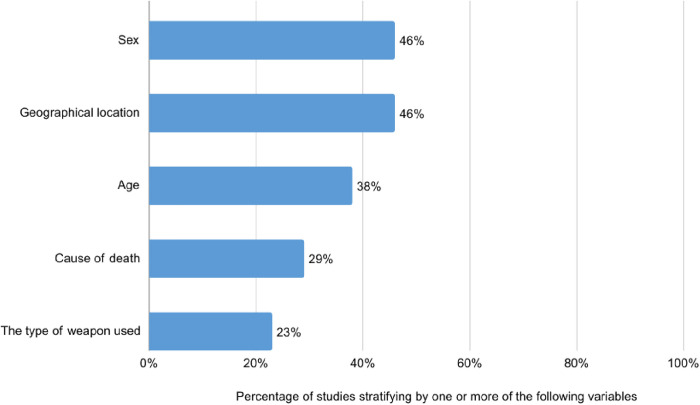
Stratification of mortality data in the studies reviewed.

Some studies presented disaggregated results. Mortality rates were stratified by sex in 46% (32/69) of the studies, by geographical location in 46% (32/69), by age in 38% (26/69), by cause of death in 29% (20/69), and by type of weapon in 23% (16/69) (Fig 3). Only 8 studies reported the alleged perpetrators.

#### 3.1.4 Challenges in obtaining data.

Possible sources of respondent bias were discussed in 15 (22%) studies. Authors expected concerns about data confidentiality and sharing, lack of trust, cultural and religious beliefs to affect death reporting. They also expected under-reporting of children’s, women’s, and combatant deaths. Survivor bias was mentioned in 11 studies (16%), recall bias in 10 studies (15%), and concerns about the quality or impartiality of health information systems were mentioned in 12 studies (17.4%). Insecurity and geographical inaccessibility limited data collection in 12 studies (17.4%). Logistic challenges (8/69, 11.6%) included internet and electricity problems, poor testing capacity for COVID-19, and low forensic lab standards. Political reasons, such as lack of government permission, were explicitly mentioned as hindering data collection in 2 studies (3%).

### 3.2 Databases reporting conflict related deaths

A total of 23 databases were included in the study (Table A in S4 Text). Of these, 13 (57%) were affiliated with non-governmental, non-profit independent organizations, foundations, or centers. Five (22%) were affiliated with academic institutions, 3 (13%) with corporations, and 1 (4%) with governmental institutions. The affiliation of 1 database was unclear. All databases reported retrospective data, with 21 focusing on direct conflict-related deaths and 2 reporting both direct deaths and deaths from other causes that may be exacerbated by war. Most focused on passive surveillance of media reports, government and public sources; others used modelling. The target population of the databases were civilians (6/23, 26%), combatants (1/23, 4%), civilians and combatants (14/23, 61%), while the remainder was unspecified (2/23, 9%). Half of the databases (11/23, 48%) relied solely on secondary data, while only two database (9%) utilized primary data only. Seven databases (30%) used a combination of primary and secondary data, while the type of data used was unclear in 3 databases (13%).

The media was a source of information in 14 (61%) databases. Twenty-one databases (91%) stratified mortality data by geographical location, and 20 (87%) by alleged perpetrator. Table B and Table C in S4 Text provides details on the methodologies used by these databases.

Data verification methods reported by some of the databases included cross-referencing incidents across multiple sources (10/23, 44%), verifying the incidents and the deaths by more than one researcher (1/23, 4%), by video, visuals, and/or satellite imaging (5/23, 22%), or using lists of victims (2/23, 9%). Some databases continuously update their records as new information become available (6/23, 26%).

Some databases discussed possible sources of bias expected to affect results. These included concerns about data quality and reliability (6/23, 26%), variability in reporting the number of deaths by different sources (6/23, 26%), expected bias of media and eyewitnesses (6/23, 26%), restricted access to reliable information and concerns about lack of transparency (5/23, 22%) as well as overlapping information and double counting (1/23, 4%). There were also some practical challenges affecting the results including language barriers in interpreting and translating information and restrictions to English media (3/23, 13%), difficulty in distinguishing civilians from combatants (3/23, 13%), security concerns and geographical inaccessibility to conflict areas (5/23, 22%), electricity and internet cut-outs in conflict areas (1/23, 4%), and definitional limitations (1/23, 4%).

### 3.4 Government websites reporting conflict related deaths

The Palestinian Central Bureau of Statistics and the Palestinian Ministry of Health was the only source of data on conflict-related deaths we found among all Government websites reviewed in the then study countries (Table 4).

**Table 4:** The governmental sources of mortality in the studied countries.

Country	Governmental mortality sources	Reporting of conflict related deaths on websites
Occupied Palestinian territory	Ministry of Health website Palestinian Central Bureau of Statistics (PCBS)	Yes *(Total numbers)*
Lebanon	Ministry of Health website The Central Administration of Statistics-Lebanon (CAS) Ministry of Interior and Municipalities	Not reporting
Pakistan	Pakistan Bureau of Statistics	
Libya	Ministry of Health	
Iraq	Central Statistical Organization (CSO) under the Iraqi Ministry of Planning	
Syria	Central Bureau of Statistics	
Yemen	Central Statistical Organization	
Afghanistan	National Statistic and Information Authority	
Somalia	Somali National Bureau of Statistics	
Sudan	Central Bureau of Statistics	Website was not functional*

a Last accessed on 5 November 2024.

### 3.5 Nutrition and mortality surveys

A total of 114 nutrition and mortality surveys were retrieved. In this subset of nutrition and mortality surveys, the most represented countries were Yemen (51/144, 45%) and Sudan (26/114, 23%) and the least represented were Syria (1/144) and Iraq (1/144). We found no surveys from Libya, Lebanon, or the occupied Palestinian territory. UNICEF (71/114, 62%) and Action Against Hunger (43/114, 38%) were the most involved organizations, and UNICEF (58/114, 51%) and the European Civil Protection and Humanitarian Aid Operations (34/114, 30%) were the main funders. Further details on the characteristics of these survey reports are available in Table A of S5 Text.

All survey designs were retrospective, derived from household surveys focused on nutritional assessment and mortality, and they all employed two-stage cluster sampling. Almost all (113/114, 99%) were sub-national in scope, applied SMART methods, and targeted the entire population (110/114, 97%). None of the surveys mentioned the alleged perpetrator. For verification, the most common method was confirmation by supervisors (31/114, 27%). Most surveys (108/114, 95%) stratified mortality by age and almost half (56/114, 49%) by sex. Three quarters of the survey reports (34/47, 72%) mentioned security concerns preventing them from accessing certain areas or loss of data because of security issues. A detailed breakdown of the methods reported outcomes, and challenges of these mortality surveys is presented in Table B of S5 Text.

## 4. Discussion

This scoping review synthetized and mapped characteristic, methods and reported outcomes of peer-reviewed studies, databases, Government websites, and a subset of nutrition and mortality surveys estimating conflict-related deaths in ten countries of the WHO Eastern Mediterranean Region between January 2004 and June 2024.

Some countries were better represented in the peer-reviewed literature (e.g., Iraq), and others in the subset of nutrition and mortality surveys reviewed (e.g., Yemen, Somalia and Sudan). Only in the occupied Palestinian territory we found data on conflict related deaths on the Palestinian Central Bureau of Statistics and the Ministry of Health website.

There was substantial variation in the number of peer-reviewed studies on conflict-related deaths across different countries. Iraq had the highest number of studies both in terms of individual country studies and multi country studies. Possible reasons include the global political implications of the war in Iraq and disputes among researchers about reported death tolls [21], all of which likely drew increased media and research scrutiny [11,22–24]. While Iraq attracted considerable attention in the peer-reviewed literature, the opposite is true for other countries (e.g., Libya in both the peer-reviewed literature and the nutrition and mortality surveys). Lack of research funding could also have been an issue given that only a little more than half of the peer-reviewed studies (38/69, 55%), reported a source of funding. We did not find any study in Arabic and only a minority of the authors were from the Region. Given the sensitivity of the topic, one could expect local authors to be hesitant to publish their findings in both national or international journals. It remains unclear whether the limited contribution of local researchers was due to the fear of retaliation for publishing politically sensitive data or the broader phenomenon of Northern dominance. It is worth noting that some studies included researchers with Arabic names, yet these individuals were associated with institutions located outside the region.

Only 26% of the peer-reviewed studies utilized governmental sources of information. Possible reasons include weaknesses in civil and vital registration systems in the region [25] and limited published data as found in our review of governmental sources. When available and of good quality, Government sources are used as shown in the case of the occupied Palestinian territory [26–28].

To account for the challenges of accessibility and/or survivor bias, some researchers have started using alternative methods to estimate conflict-related data. These methods include satellite imagery, web-based surveys, capture‑recapture analysis, and small-area estimation. We found evidence on this in our study. Two studies conducted in Yemen used satellite imagery [29,30], capture-recapture analysis was employed in three of the reviewed studies [31–33], and small-area estimation in one study [34]. These methods are not without their limitations as highlighted in the studies and their use should assessed in the context of the study country.

The number of peer-reviewed studies using data from conflict-related death databases highlighted the importance of this source of information. We reviewed 22 databases and observed some shortcomings. There were issues of transparency in some databases. For example, the funding source was unclear for seven databases, data sources were vague for three, and there was a lack of clarity regarding humanitarian organizations, research institutions, or media outlets used as sources as already observed by Seybolt (2002) [35]. Additionally, it was previously noted that these databases often under-report death rates [15,36,37], a problem particularly highlighted in media-based databases.

The Centre for Research on the Epidemiology of Disasters (CRED) used to maintain a database of nutrition and mortality surveys on nutrition and mortality but has been discontinued. Other sources of survey compilations were mentioned in papers reviewing quality of mortality surveys [38,39] which were not accessible online at the time this study was conducted. The extensive use of databases based on passive surveillance in the peer-reviewed literature suggests a strong interest in mortality data in conflict settings, especially longitudinal data. It seems therefore important to explore ways of compiling such surveys to make them more easily accessible and increase their use. Indeed, our search strategy only captured the most readily accessible with the idea that these are likely to be the ones researchers and other users can most easily access.

Numerous studies have been conducted on deaths of military personnel from foreign countries such as the UK, USA and Canada operating in the region [40–45]. However, we did not find any study on mortality among local military forces. The absence of such studies likely reflects the political sensitivity of the issue.

While this scoping review provides a valuable overview of the current state of research on conflict -related deaths in the EMR, it is important to acknowledge its limitations. First, the search of the peer-reviewed literature was limited to three databases (PubMed, Web of Science, and the Global Index Medicus), which, albeit major ones, may have failed to retrieve some relevant studies. Second, the search relied on specific keywords, which could have inadvertently excluded studies that used different terminology, particularly in the wide area of indirect deaths. Third, our search for nutrition and mortality surveys was limited to publicly accessible sources on the websites of key organizations. We did not contact operational actors in the region to request unpublished or internal survey data or used advanced searching tools such as web scraping. As a result, the nutrition and mortality surveys data included in this review represent only a subset of the total surveys conducted. Fourth, the extraction process involved a single reviewer, although any uncertainties or disagreements were addressed through team discussion to ensure consensus. Fifth, this being a scoping review, we did not assess the quality of the methods used. Finally, we did not look at sub-national coverage and areas, within a country, that may not have been covered.

## 5. Conclusion

This scoping review identified a wide spectrum of methods used to estimate conflict-related deaths in 10 countries of the WHO Eastern Mediterranean Region and important variations in the volume of work in different countries affected by armed conflict in the past 20 years. The use of databases based on passive surveillance was prominent in the peer-reviewed literature possibly due to the lack of alternative readily accessible longitudinal data from primary data sources or civil registration and vital statistics for countries in conflict. Estimating deaths due to armed conflict is notoriously difficult since in most cases, civil registration and vital statistics systems were not reliable already before conflict or if they were, they usually become less and less complete as the conflict progresses. Regularly performed well designed mortality survey are key to monitor mortality in conflict affected settings and the resulting reports should be easily accessible, ideally compiled in a repository. There is a need to validate existing methods and train humanitarian workers in epidemiology. Studies thus far have often relied on foreign expertise. Greater involvement of local researchers and civil society organization (already done but needs to become routine) will help building capacity and identifying methods which are operational in different context. Establishing prospective mortality surveillance systems in relatively stable regions within conflict-affected countries is another option but it requires sustained supervision of data collectors and stable denominators which makes it is more feasible in organised camps for internally displaced people and refugees. All databases reporting on conflict-related deaths should maintain transparency regarding their sources, funding, and affiliations to uphold impartiality and credibility. There have been many calls for improving methods to estimate conflict-related deaths over the past 20 years and various initiatives launched at different points in time to that end. Some progress has been made but we still struggle to systematically measure this critical indicator. It is high time to stem the tide and we hope that the findings of this review, can be a small step in that direction.

## Supporting information

S1 Checklist Preferred Reporting Items for Systematic reviews and Meta-Analyses extension for Scoping Reviews (PRISMA-ScR) Checklist. (DOCX)

S1 Text Used search strategy for peer-reviewed articles. (DOCX)

S2 Text Extraction form of the scoping review. (DOCX)

S3 Text Details about the peer-reviewed articles. (DOCX)

S4 Text Details about the conflict-related deaths databases. (DOCX)

S5 Text Details about the nutrition and mortality surveys. (DOCX)

S1 Data The details of included peer-reviewed articles, nutrition and mortality surveys, and databases. (XLSX)
