# Supplementary material for: Measuring conflict related mortality in ten countries of the WHO Eastern Mediterranean Region (2004–2024): A scoping review
Source: PLOS Glob Public Health. 2025 Nov 11;5(11):e0005465. doi: 10.1371/journal.pgph.0005465 (PMC12604791; doi:10.1371/journal.pgph.0005465)
Supplement: S1 Text — (DOCX) [file pgph.0005465.s002.docx]

**S1 Text: The JBI three-step search strategy and the used search strategy for peer-reviewed articles**

**Box A. The JBI three-step search strategy:**

First, the research team, in consultation with a librarian, developed the initial search strategy focusing on three concepts: deaths, conflict, and EMR countries and incorporated a combination of MeSH terms and text words. These terms were used to conduct a preliminary search in PubMed and Web of Science. Using Atlas.ti software, we analysed the text words in the titles and abstracts of retrieved articles to identify relevant keywords and index terms and modify the search terms accordingly. Second, these terms were then employed for a more comprehensive search across all three databases. Third, we examined the reference lists of identified reviews to uncover additional relevant articles.

**Table A. The used search strategy for peer-reviewed articles**

| Database | PubMed |
| --- | --- |
| Date of search | 31/07/2024 |
| Search query | ((“Mediterranean Region” OR “Eastern Mediterranean” OR Eastern-Mediterranean OR EMRO OR EMR OR “Middle East” OR “Middle Eastern” OR Arab OR “North Africa” OR “North African” OR Levant OR Maghreb OR “Horn of Africa” OR Iraq OR Iraqi OR Afghanistan OR Afghan OR Lebanon OR Lebanese OR Libya OR Libyan OR Pakistan OR Pakistani OR Palestine OR Palestinian OR “West Bank” OR Gaza OR Somalia OR Somalian OR Sudan OR Sudanese OR Syria OR Syrian OR Yemen OR Yemeni) AND (("Conflict Death"[tiab:~5] OR "Conflict Deaths"[tiab:~5] OR "Conflict Mortality"[tiab:~5] OR "Conflict Mortalities"[tiab:~5] OR "Conflict Fatality"[tiab:~5] OR "Conflict Fatalities"[tiab:~5] OR “Conflict Killed"[tiab:~5] OR “Conflict Killing"[tiab:~5]) OR ("Conflicts Death"[tiab:~5] OR "Conflicts Deaths"[tiab:~5] OR "Conflicts Mortality"[tiab:~5] OR "Conflicts Mortalities"[tiab:~5] OR "Conflicts Fatality"[tiab:~5] OR "Conflicts Fatalities"[tiab:~5] OR “Conflicts Killed"[tiab:~5] OR “Conflicts Killing"[tiab:~5]) OR ("War Death"[tiab:~5] OR "War Deaths"[tiab:~5] OR "War Mortality"[tiab:~5] OR "War Mortalities"[tiab:~5] OR "War Fatality"[tiab:~5] OR "War Fatalities"[tiab:~5] OR “War Killed"[tiab:~5] OR “War Killing"[tiab:~5]) OR ("Violence Death"[tiab:~5] OR "Violence Deaths"[tiab:~5] OR "Violence Mortality"[tiab:~5] OR "Violence Mortalities"[tiab:~5] OR "Violence Fatality"[tiab:~5] OR "Violence Fatalities"[tiab:~5] OR “Violence Killed"[tiab:~5] OR “Violence Killing"[tiab:~5]) OR ("Violent Death"[tiab:~5] OR “Violent Deaths"[tiab:~5] OR "Violent Mortality"[tiab:~5] OR "Violent Mortalities"[tiab:~5] OR "Violent Fatality"[tiab:~5] OR "Violent Fatalities"[tiab:~5] OR “Violent Killed"[tiab:~5] OR “Violent Killing"[tiab:~5]) OR ("Battle Death"[tiab:~5] OR “Battle Deaths"[tiab:~5] OR "Battle Mortality"[tiab:~5] OR "Battle Mortalities"[tiab:~5] OR "Battle Fatality"[tiab:~5] OR "Battle Fatalities"[tiab:~5] OR “Battle Killed"[tiab:~5] OR “Battle Killing"[tiab:~5]) OR ("Insurgency Death"[tiab:~5] OR “Insurgency Deaths"[tiab:~5] OR "Insurgency Mortality"[tiab:~5] OR "Insurgency Mortalities"[tiab:~5] OR "Insurgency Fatality"[tiab:~5] OR "Insurgency Fatalities"[tiab:~5] OR “Insurgency Killed"[tiab:~5] OR “Insurgency Killing"[tiab:~5]) OR ("Terrorism Death"[tiab:~5] OR “Terrorism Deaths"[tiab:~5] OR "Terrorism Mortality"[tiab:~5] OR "Terrorism Mortalities"[tiab:~5] OR "Terrorism Fatality"[tiab:~5] OR "Terrorism Fatalities"[tiab:~5] OR “Terrorism Killed"[tiab:~5] OR “Terrorism Killing"[tiab:~5]) OR ("Terrorist Death"[tiab:~5] OR “Terrorist Deaths"[tiab:~5] OR "Terrorist Mortality"[tiab:~5] OR "Terrorist Mortalities"[tiab:~5] OR "Terrorist Fatality"[tiab:~5] OR "Terrorist Fatalities"[tiab:~5] OR “Terrorist Killed"[tiab:~5] OR “Terrorist Killing"[tiab:~5]))) |
| Filters | Publication Date: from 2004/1/1 - 2024/6/1 |
| Number of hits | 329 |
| Database | **Web of Science** |
| Date of search | 1/8/2024 |
| Search query | ((TI=(“Mediterranean Region” OR “Eastern Mediterranean” OR Eastern-Mediterranean OR EMRO OR EMR OR “Middle East” OR “Middle Eastern” OR Arab OR “North Africa” OR “North African” OR Levant OR Maghreb OR “Horn of Africa” OR Iraq OR Iraqi OR Afghanistan OR Afghan OR Lebanon OR Lebanese OR Libya OR Libyan OR Pakistan OR Pakistani OR Palestine OR Palestinian OR “West Bank” OR Gaza OR Somalia OR Somalian OR Sudan OR Sudanese OR Syria OR Syrian OR Yemen OR Yemeni)) AND TI=((Conflict OR Conflicts OR War OR Violence OR Violent OR Terrorism OR Terrorist OR Battle OR Insurgency) NEAR/5 (Death OR Deaths OR Mortality OR Mortalities OR Fatality OR Fatalities OR Killed OR Killing))) OR ((AB=(“Mediterranean Region” OR “Eastern Mediterranean” OR Eastern-Mediterranean OR EMRO OR EMR OR “Middle East” OR “Middle Eastern” OR Arab OR “North Africa” OR “North African” OR Levant OR Maghreb OR “Horn of Africa” OR Iraq OR Iraqi OR Afghanistan OR Afghan OR Lebanon OR Lebanese OR Libya OR Libyan OR Pakistan OR Pakistani OR Palestine OR Palestinian OR “West Bank” OR Gaza OR Somalia OR Somalian OR Sudan OR Sudanese OR Syria OR Syrian OR Yemen OR Yemeni)) AND AB=((Conflict OR Conflicts OR War OR Violence OR Violent OR Terrorism OR Terrorist OR Battle OR Insurgency) NEAR/5 (Death OR Deaths OR Mortality OR Mortalities OR Fatality OR Fatalities OR Killed OR Killing))) |
| Filters | Publication Date: 2004-01-01 to 2024-06-01 / NOT retracted publication or meeting abstract |
| Number of hits | 517 |
| Database | **Global Index Medicus** |
| Date of search | 1/8/2024 |
| Search query | ((TI:(“Mediterranean Region” OR “Eastern Mediterranean” OR Eastern-Mediterranean OR EMRO OR EMR OR “Middle East” OR “Middle Eastern” OR Arab OR “North Africa” OR “North African” OR Levant OR Maghreb OR “Horn of Africa” OR Iraq OR Iraqi OR Afghanistan OR Afghan OR Lebanon OR Lebanese OR Libya OR Libyan OR Pakistan OR Pakistani OR Palestine OR Palestinian OR “West Bank” OR Gaza OR Somalia OR Somalian OR Sudan OR Sudanese OR Syria OR Syrian OR Yemen OR Yemeni)) AND TI:((Conflict OR Conflicts OR War OR Violence OR Violent OR Terrorism OR Terrorist OR Battle OR Insurgency) AND (Death OR Deaths OR Mortality OR Mortalities OR Fatality OR Fatalities OR Killed OR Killing))) OR ((AB:(“Mediterranean Region” OR “Eastern Mediterranean” OR Eastern-Mediterranean OR EMRO OR EMR OR “Middle East” OR “Middle Eastern” OR Arab OR “North Africa” OR “North African” OR Levant OR Maghreb OR “Horn of Africa” OR Iraq OR Iraqi OR Afghanistan OR Afghan OR Lebanon OR Lebanese OR Libya OR Libyan OR Pakistan OR Pakistani OR Palestine OR Palestinian OR “West Bank” OR Gaza OR Somalia OR Somalian OR Sudan OR Sudanese OR Syria OR Syrian OR Yemen OR Yemeni)) AND AB:((Conflict OR Conflicts OR War OR Violence OR Violent OR Terrorism OR Terrorist OR Battle OR Insurgency) AND (Death OR Deaths OR Mortality OR Mortalities OR Fatality OR Fatalities OR Killed OR Killing))) |
| Filters used | Publication Date: 2004 to 2024 |
| Number of hits | 5 |
