# Supplementary material for: Measuring conflict related mortality in ten countries of the WHO Eastern Mediterranean Region (2004–2024): A scoping review
Source: PLOS Glob Public Health. 2025 Nov 11;5(11):e0005465. doi: 10.1371/journal.pgph.0005465 (PMC12604791; doi:10.1371/journal.pgph.0005465)
Supplement: S2 Text — (DOCX) [file pgph.0005465.s003.docx]

**S2 Text: Extraction form of the scoping review**

Study Characteristics

| Field | Details |
| --- | --- |
| Study ID |  |
| Title |  |
| Author(s) |  |
| Author’s Affiliation |  |
| Country |  |
| Is the first author affiliated with an institution from the region? | Yes / No |
| Are any other authors affiliated with an institution from the region? | Yes / No |
| Publication Year |  |
| Publisher |  |
| Funder |  |
| Language |  |

Study Scope

| Scope | Details |
| --- | --- |
| Country |  |
| Scope of Mortality | 1. Direct Deaths  2. Indirect Deaths  3. Both |
| Type of Mortality Data | 1. Primary  2. Secondary  3. Mixed |
| Study Design | 1. Prospective  2. Retrospective  3. Prospective-Retrospective |

Data Sources

| Source of Data | Details |
| --- | --- |
| Governmental Report | Yes / No  Name: __________ |
| Humanitarian and research organizations | Yes / No  Name: __________ |
| Conflict-related deaths databases | Yes / No  Name: __________ |
| Primary Data Collection (e.g., Household Survey) | Yes / No  Name: __________ |
| Others | Yes / No  Name: __________ |

Study Population and Location

| Field | Details |
| --- | --- |
| Sampling Method |  |
| Targeted Population |  |
| Inclusion Criteria |  |
| Exclusion Criteria |  |
| Population Type | 1. Civilian  2. Non-Civilian  3. Both  4. Not Specified |
| Geographical Location | 1. National  2. Sub-national |

Data Analysis

| Data Analysis Methods | Details |
| --- | --- |
| Types of Analysis | 1. Descriptive Statistics  2. Inferential Statistics  3. Time Series Analysis  4. Spatial Analysis  5. Qualitative Analysis  6. Other |
| Study Duration |  |
| Period Studied |  |
| Data Verification Methods |  |

Reported Outcomes

| Outcome Measures | Details |
| --- | --- |
| Total Number of Deaths | Yes / No |
| Overall Mortality Rate | Yes / No  Specify: __________ |
| Excess Mortality (Number/Rate) | Yes / No  Specify: __________ |
| Cause-Specific Mortality (e.g., Conflict/Violence/Injury/Detention/MMR) | Yes / No  Specify: __________ |
| Proportionate Mortality (e.g., Conflict/Violent Deaths) | Yes / No  Specify: __________ |
| Age-Specific (e.g., IMR) | Yes / No  Specify: __________ |
| Morbidity and Burden of Disease (e.g., YLL/YLS/DALY/Disability) | Yes / No  Specify: __________ |
| Other |  |

Data Stratification

| Stratification Variable | Options |
| --- | --- |
| Cause of Death | Yes / No |
| Type of Weapon Used | Yes / No |
| Geographical Location | Yes / No |
| Stratification by Age | Yes / No |
| Stratification by Sex | Yes / No |

Definitions

| Definition | Details |
| --- | --- |
| Conflict-Related Death |  |
| Direct Deaths |  |
| Indirect Deaths |  |

Additional Information

| Field | Details |
| --- | --- |
| Alleged Perpetrator Mentioned | 1. Yes  2. No  3. Not Applicable |
| Challenges in Obtaining Data |  |
