# Supplementary material for: Measuring conflict related mortality in ten countries of the WHO Eastern Mediterranean Region (2004–2024): A scoping review
Source: PLOS Glob Public Health. 2025 Nov 11;5(11):e0005465. doi: 10.1371/journal.pgph.0005465 (PMC12604791; doi:10.1371/journal.pgph.0005465)
Supplement: S3 Text — (DOCX) [file pgph.0005465.s004.docx]

**S3 Text: Details about the peer-reviewed articles**
 **Table A. The affiliation of first authors by country and institutions (N=69)**

| **First Author Affiliation** | **Author's Affiliations by Country** | **n** | **%** | **Author's Institutional Affiliations** | **n** | **%** |
| --- | --- | --- | --- | --- | --- | --- |
| First author affiliated with an EMR institution (N=15) | Iraq | 5 | 33.3 | Al Mustansiriya University | 3 | 60 |
|  |  |  |  | University of Anbar | 1 | 20 |
|  |  |  |  | World Health Organization | 1 | 20 |
|  | Lebanon | 2 | 13.3 | American University of Beirut | 1 | 50 |
|  |  |  |  | University of Balamand | 1 | 50 |
|  | Libya | 3 | 20.0 | University of Tripoli | 3 | 100 |
|  | Pakistan | 1 | 6.7 | The Aga Khan University | 1 | 100 |
|  | Somalia | 1 | 6.7 | World Health Organization | 1 | 100 |
|  | Syria | 2 | 13.3 | Bab Al-Hawa Hospital | 1 | 50 |
|  |  |  |  | Médecins Sans Frontières | 1 | 50 |
|  | Yemen | 1 | 6.7 | Hadhramout University College of Medicine | 1 | 100 |
| First author affiliated with an institution outside EMR (N=54) | Belgium | 5 | 9.3 | Médecins Sans Frontières | 1 | 20 |
|  |  |  |  | Université catholique de Louvain | 4 | 80 |
|  | Canada | 2 | 3.7 | Simon Fraser University | 1 | 50 |
|  |  |  |  | University of Toronto | 1 | 50 |
|  | France | 3 | 5.6 | Centre Maurice Halbwachs | 1 | 33.3 |
|  |  |  |  | Epicentre | 2 | 66.7 |
|  | Germany | 3 | 5.6 | Heidelberg University | 2 | 66.7 |
|  |  |  |  | University of Mannheim | 1 | 33.3 |
|  | Israel | 1 | 1.9 | Ben-Gurion University of the Negev | 1 | 100 |
|  | New Zealand | 1 | 1.9 | University of Otago Wellington | 1 | 100 |
|  | Norway | 1 | 1.9 | Hedmark University of Applied Sciences | 1 | 100 |
|  | Switzerland | 1 | 1.9 | Small Arms Survey | 1 | 100 |
|  | UK | 10 | 18.5 | King's College London | 2 | 20 |
|  |  |  |  | London School of Hygiene and Tropical Medicine | 6 | 60 |
|  |  |  |  | Royal Holloway University of London | 1 | 10 |
|  |  |  |  | University of London | 1 | 10 |
|  | USA | 27 | 50.0 | Beth Israel Deaconess Medical Center | 1 | 3.7 |
|  |  |  |  | Centers for Disease Control and Prevention | 4 | 14.8 |
|  |  |  |  | Columbia University | 1 | 3.7 |
|  |  |  |  | David Geffen School of Medicine | 1 | 3.7 |
|  |  |  |  | George Mason University | 1 | 3.7 |
|  |  |  |  | Harvard University | 2 | 7.4 |
|  |  |  |  | Hospital for Sick Children | 1 | 3.7 |
|  |  |  |  | Institute for Health Metrics and Evaluation | 1 | 3.7 |
|  |  |  |  | International Society for Infectious Diseases | 1 | 3.7 |
|  |  |  |  | James Madison University | 2 | 7.4 |
|  |  |  |  | Johns Hopkins Bloomberg School of Public Health | 3 | 11.1 |
|  |  |  |  | Lehigh Valley Health Network | 1 | 3.7 |
|  |  |  |  | New York University | 1 | 3.7 |
|  |  |  |  | Rice University | 1 | 3.7 |
|  |  |  |  | University of California Davis Department of Surgery | 1 | 3.7 |
|  |  |  |  | University of Washington | 5 | 18.5 |

**Table B. The funding institutions for the included peer-reviewed studies**

| **Funding Institutions** | **Frequency** |
| --- | --- |
| United Kingdom Foreign Commonwealth and Development Office (FCDO) | 5 |
| Surgeons OverSeas (SOS) | 5 |
| Bill & Melinda Gates Foundation | 4 |
| Médecins Sans Frontières (MSF) | 4 |
| European Commission | 2 |
| Johns Hopkins Bloomberg School of Public Health | 2 |
| Emirates Foundation for Philanthropy | 1 |
| Fogarty International Center | 1 |
| George Mason University Libraries Open Access Publishing Fund | 1 |
| Global Challenges Research Fund | 1 |
| International Society for Infectious Diseases | 1 |
| Laboratory for Analytic Sciences (LAS) | 1 |
| Massachusetts Institute of Technology (MIT) | 1 |
| National Science Foundation (NSF) | 1 |
| Sigrid Rausing Trust | 1 |
| Small Arms Survey | 1 |
| Social Sciences and Humanities Research Council of Canada | 1 |
| Supported by the United Nations Development Group Iraq Trust Fund | 1 |
| The UK Department for International Development funded the complex emergency database project | 1 |
| United States Agency for International Development (USAID) | 1 |
| US Department of State Bureau of Population, Refugees and Migration | 1 |
| US Fund for Countdown to 2030 | 1 |
| US National Institutes of Health | 1 |
| World Health Organization (WHO) | 1 |
| Amazon Research Award | 1 |
| Australian Agency for International Development (AusAID) | 1 |
| Centers for Disease Control and Prevention (CDC) | 1 |
| None /Not mentioned | 38 |

**Table C. The publisher of the included peer-reviewed studies**

| **Publisher** | **Frequency** | **Percent** |
| --- | --- | --- |
| BMC Conflict and Health | 8 | 11.6 |
| Lancet | 7 | 10.1 |
| Prehospital and Disaster Medicine | 6 | 8.7 |
| PLOS Medicine | 5 | 7.2 |
| PLOS ONE | 4 | 5.8 |
| BMJ Global Health | 3 | 4.3 |
| BMC Public Health | 2 | 2.9 |
| PLOS Global Public Health | 2 | 2.8 |
| Asian Population Studies | 1 | 1.4 |
| Disaster Medicine and Public Health Preparedness | 1 | 1.4 |
| Eastern Mediterranean Health Journal | 1 | 1.4 |
| European Journal of Sociology | 1 | 1.4 |
| European Journal on Criminal Policy and Research | 1 | 1.4 |
| Injury | 1 | 1.4 |
| Int J Public Health | 1 | 1.4 |
| International Journal of Epidemiology | 1 | 1.4 |
| International Journal of Infectious Diseases | 1 | 1.4 |
| International Journal of Injury Control and Safety Promotion | 1 | 1.4 |
| JAMA | 1 | 1.4 |
| Journal of Peace Research | 1 | 1.4 |
| Journal of the International Society for Burn Injuries | 1 | 1.4 |
| Lancet Glob Health | 1 | 1.4 |
| Medicine, Conflict and Survival | 1 | 1.4 |
| Patterns of Violence against Ethnic Enclaves | 1 | 1.4 |
| PeerJ | 1 | 1.4 |
| Public Health Action | 1 | 1.4 |
| Research and Politics | 1 | 1.4 |
| Saudi Medical Journal | 1 | 1.4 |
| Social Indicators Research | 1 | 1.4 |
| Studies in Conflict & Terrorism | 1 | 1.4 |
| The Annals of Applied Statistics | 1 | 1.4 |
| The European Journal of Public Health | 1 | 1.4 |
| The Journal of Infection in Developing Countries | 1 | 1.4 |
| The Journal of TRAUMAÂ® Injury, Infection, and Critical Care | 1 | 1.4 |
| The New England Journal of Medicine | 1 | 1.4 |
| Transactions on Ecology and the Environment | 1 | 1.4 |
| Trauma Surgery and Acute Care Open | 1 | 1.4 |
| African Journal of Emergency Medicine | 1 | 1.4 |
| Annals of Surgery | 1 | 1.4 |
| medRxiv | 1 | 1.4 |
| Total | 69 | 100.0 |

**Table D. Sources used in country-specific peer-reviewed studies**

|  | **Governmental reports or**  **websites** | | **Humanitarian and Research Organizations** | | **Conflict-related deaths databases** | | **Primary data collection** | | **Others** | |
| --- | --- | --- | --- | --- | --- | --- | --- | --- | --- | --- |
|  | **N** | **Specific source** | **N** | **Specific source** | **N** | **Specific source** | **N** | **Specific source** | **N** | **Specific source** |
| **Iraq (N=25)** | 5 | -Injury Mortality Surveillance System operated by the Iraqi Ministry of Health  -Country-level surveys  -Haditha Health Vital Statistics Centre | 2 | -Committee to Protect  -Journalists Reporters Without Borders  -UNESCO  -International News Safety Institute  -International Press Institute  - Brookings Institution database | 7 | -Iraq Body Count  -iCasualties (1 study) | 12 | Household surveys | 4 | Data from previous household surveys |
| **Yemen (N=8)** | 4 | -National surveillance system (Ministry of Health)  -Aden Civil Registry office  -Disease Early Warning System | 1 | Surveys from the Complex Emergency Database | 3 | ACLED | 4 | -Key Informants  -Satellite Imagery  -Web-based mortality survey | 0 | - |
| **Syria (N=6)** | 0 | - | 1 | -Syrian Observatory for Human Rights  -Syria Shuhada Website | 4 | -Syrian Center for Statistics and Research  -Syrian Network for Human Rights  -Violation Documentation Center | 2 | Household survey | 0 | - |
| **Palestine (N=5)** | 2 | -Palestinian Health Information Centre in Gaza  -Ministry of Health hospitals  -Death Notification Office in Gaza  -Death report from the deceased’s medical file, Primary Health Care Directorate  -Israel Ministry of Foreign Affairs  -Palestinian Central Bureau of Statistics | 2 | -International Policy Institute for Counter-Terrorism  -OCHA | 4 | -B'Tselem  -Palestinian Committee on Human Rights | 0 | - | 2 | Media |
| **Sudan (N=4)** | 0 | - | 2 | -Centre for Research on the Epidemiology of Diseases’ online complex emergency database -UN’s series about Darfur -Surveys on mortality conducted by Epicentre, MSF, WHO, EPIET, CDC, WFP, FMH, UNICEF, FAO | 0 | - | 2 | Household survey | 0 | - |
| **Libya (N=3)** | 2 | -National Death Registry offices  -Ministry of State for Families of Martyrs, Injuries and Missing Persons  -Official government reports  -Local authorities in each region  -Public hospitals and isolation centres with ICU facilities | 1 | Libyan Red Crescent | 0 | - | 2 | Key informant interview | 0 | - |
| **Somalia (N=2)** | 1 | EWARN (MOH in collaboration with WHO) | 2 | Retrospective household surveys conducted by different humanitarian actors  -WHO | 1 | ACLED | 0 | - | 0 | - |
| **Pakistan (N=2)** | 0 | - | 1 | UNDESA | 1 | South-Asian Terrorism Portal | 0 | - | 1 | Media, books, journal articles, and internet databases |
| **Afghanistan (N=2)** | 1 | -Registers of the inpatient departments of all hospitals within the catchment area of the district | 1 | International Committee of the Red Cross | 0 | - | 1 | Household survey | 0 | - |
| **Multiple countries studies** | 3 | -Vital registration data  -Demographic and Health Surveys  -Pan Arab Project for Child Development surveys  -Censuses  -Country-specific surveys-sample registration systems  -Websites of the Ministries of Health | 4 | MSF health facilities, Mortality Estimation, WHO, UNICEF, UNFPA, World Bank Group, UNDP, ProMED, United Nations Inter-agency Group for Child Mortality Estimation, United Nations Inter-agency for Maternal Mortality Estimation, CDC Reproductive health surveys | 8 | -Global Burden of Disease  -Global Terrorism Database  -Syria Tracker  -Uppsala Conflict Data Program | 1 | Key informant interview | 1 | Data from previous household surveys |

**Table E. The scope of the peer-reviewed study and the targeted population per country**

|  | **Scope of the study** | | | **Targeted population** | | |
| --- | --- | --- | --- | --- | --- | --- |
|  | **Direct deaths**  **n(%)** | **Indirect deaths**  **n(%)** | **Both**  **n(%)** | **Civilians**  **n(%)** | **Both**  **n(%)** | **Not specified**  **n(%)** |
| Afghanistan | 3 (30.0) | 2) 20.0) | 5) 50.0) | 2(20.0) | 1(10.0) | 7(70.0) |
| Iraq | 12(37.5) | 4(12.5) | 16(50.0) | 14(43.8) | 6(18.8) | 12(37.5) |
| Libya | 2(22.2) | 2(22.2) | 5(55.6) | 2(22.2) | - | 7(77.8) |
| Pakistan | 2(22.2) | 2(22.2) | 5(55.6) | 3(33.3) | 1(11.1) | 5(55.6) |
| Palestine | 4(33.3) | 3(25.0) | 5(41.7) | 4(33.3) | 2(16.7) | 6(50.0) |
| Somalia | 2(22.2) | 2(22.2) | 5(55.6) | 2(22.2) | - | 7(77.8) |
| Sudan | 2(18.2) | 1(9.1) | 8(72.7) | 3(27.3) | - | 8(72.7) |
| Syria | 6(37.5) | 4(25.0) | 6(37.5) | 6(37.5) | 3(18.8) | 7(43.8) |
| Yemen | 1(7.1) | 4(28.6) | 9(64.3) | 6(42.9) | - | 8(57.1) |
| Lebanon | 1(12.5) | 3(37.5) | 4(50.0) | 3(37.5) | - | 5(62.5) |


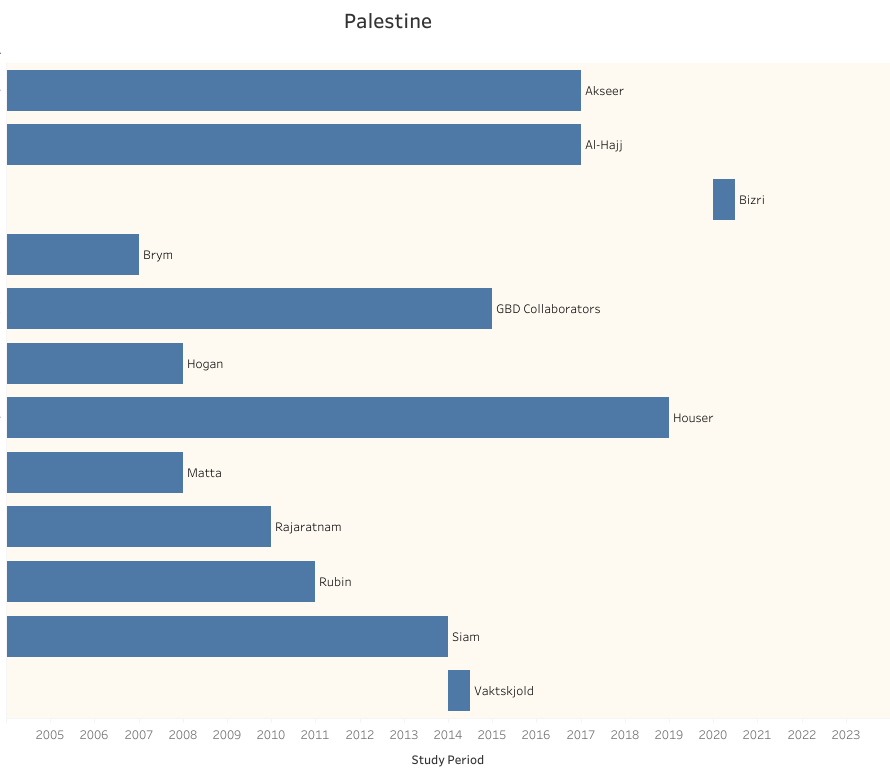


**Figure A: The periods covered by each peer-reviewed study about Palestine**


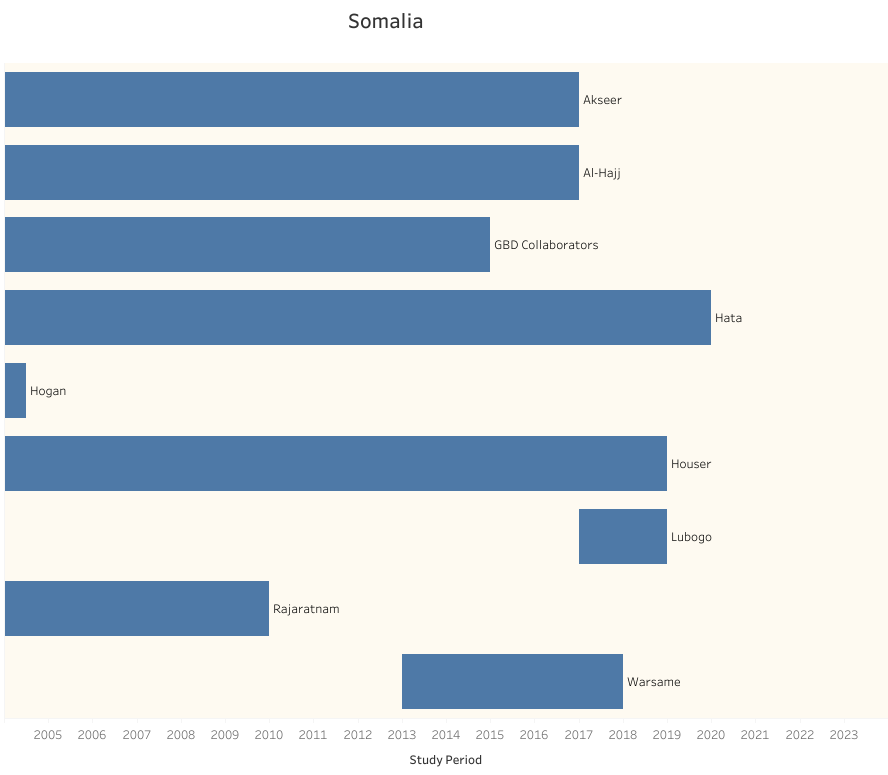


**Figure B: The periods covered by each peer-reviewed study about Somalia**


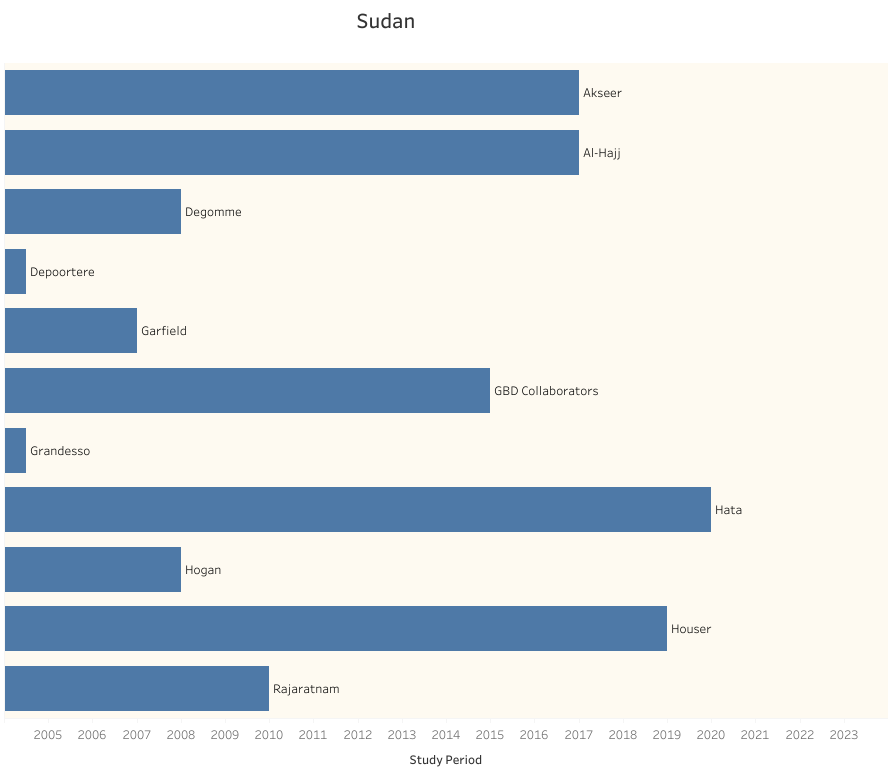


**Figure C: The periods covered by each peer-reviewed study about Sudan**


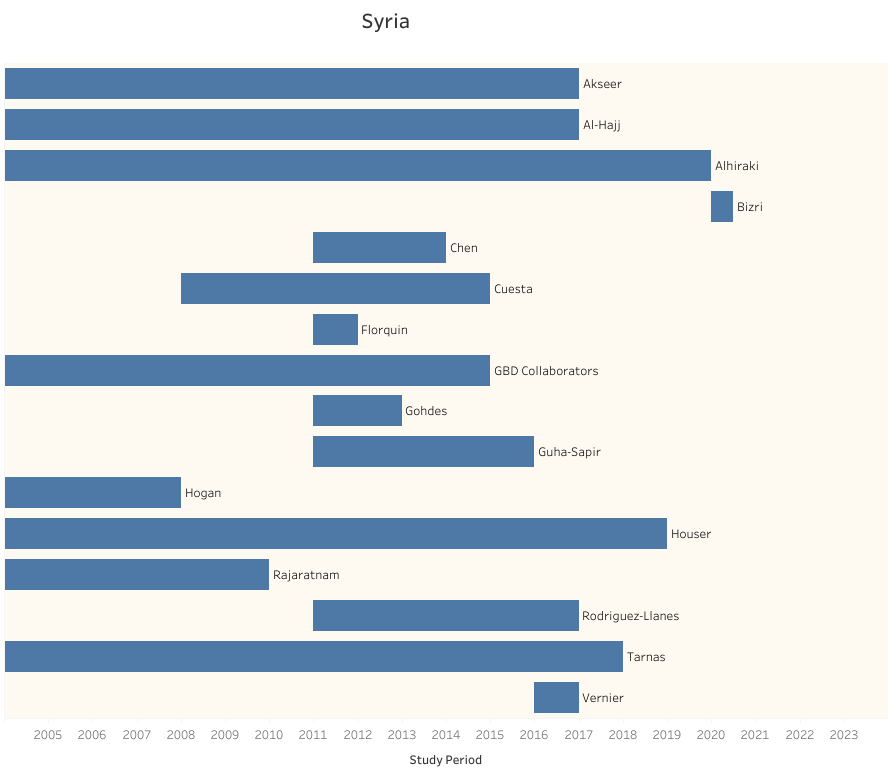


**Figure D: The periods covered by each peer-reviewed study about Syria**


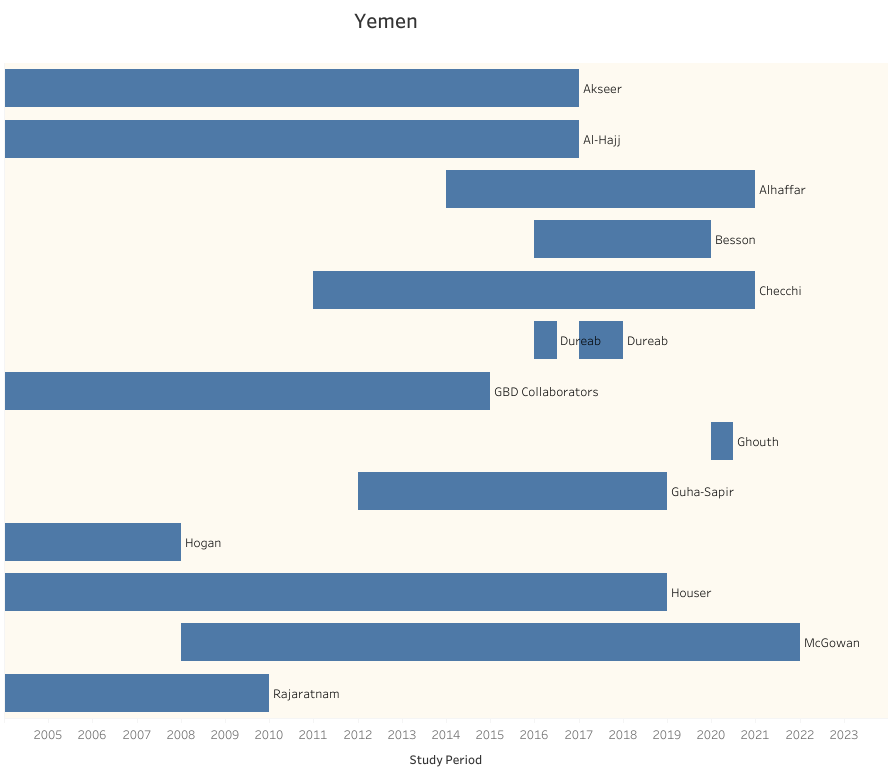


**Figure E: The periods covered by each peer-reviewed study about Yemen**


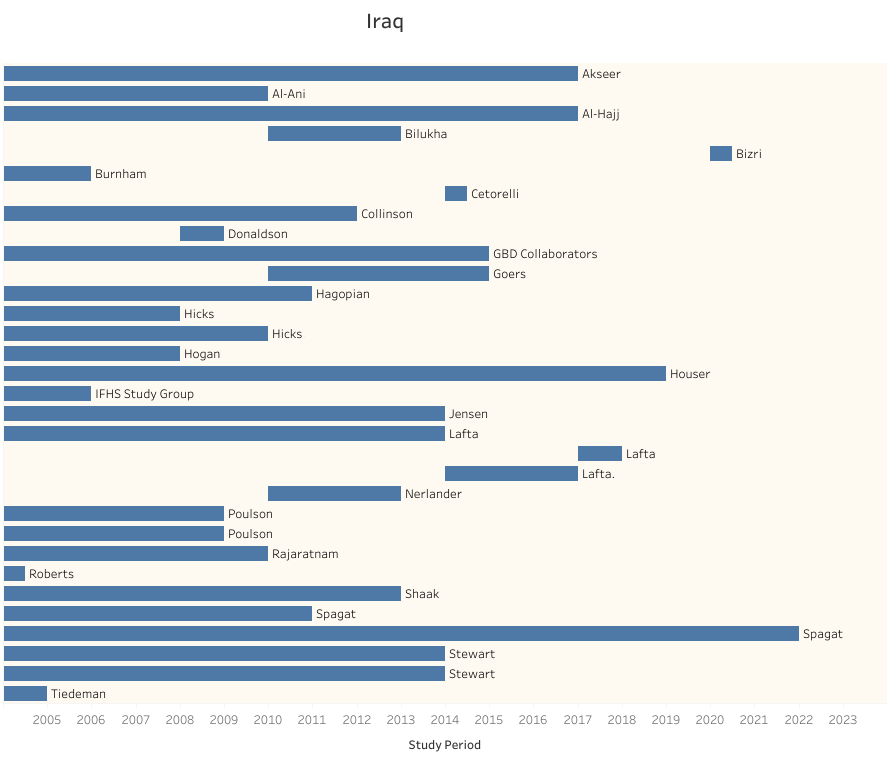


**Figure F: The periods covered by each peer-reviewed study about Iraq**


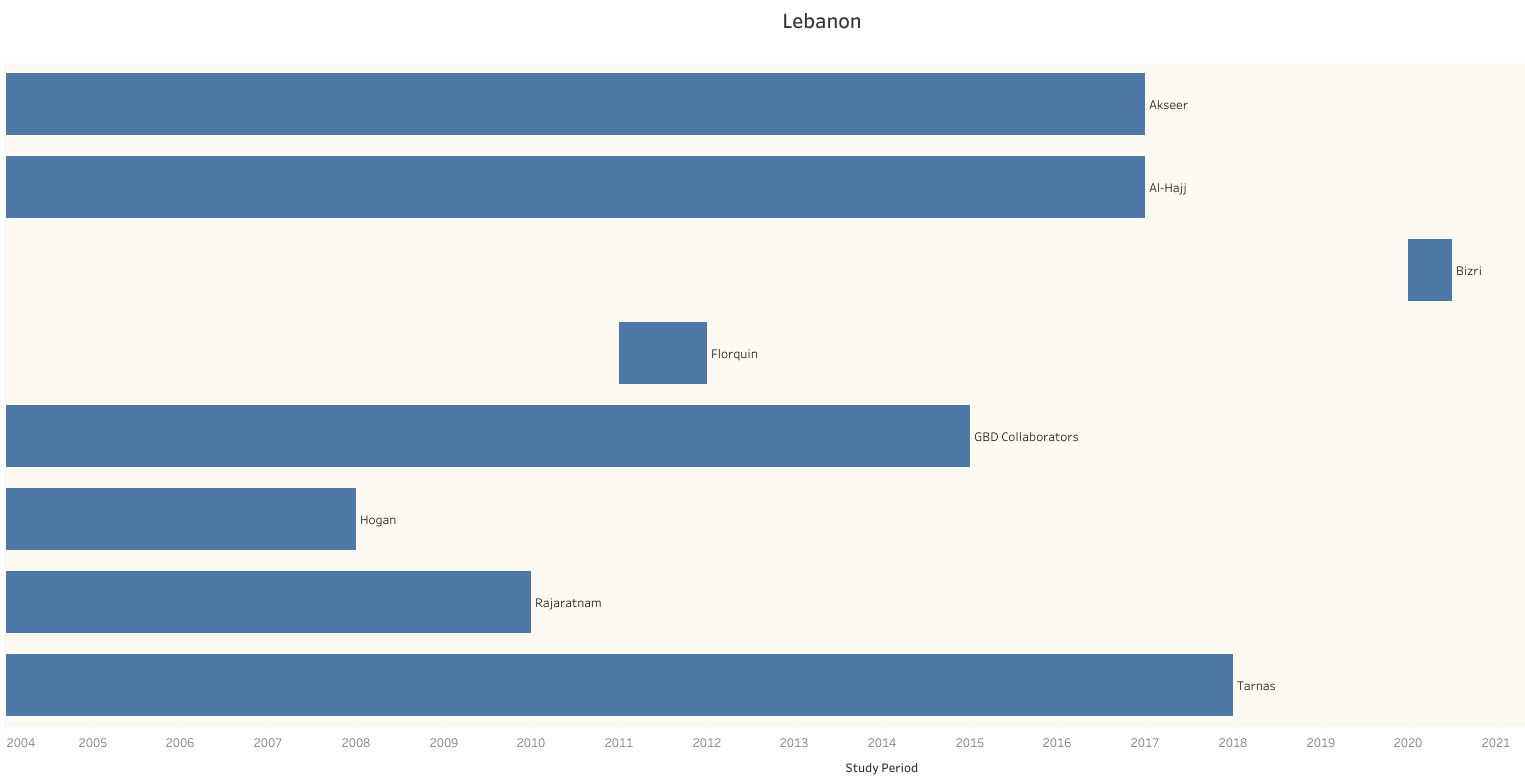


**Figure G: The periods covered by each peer-reviewed study about Lebanon**


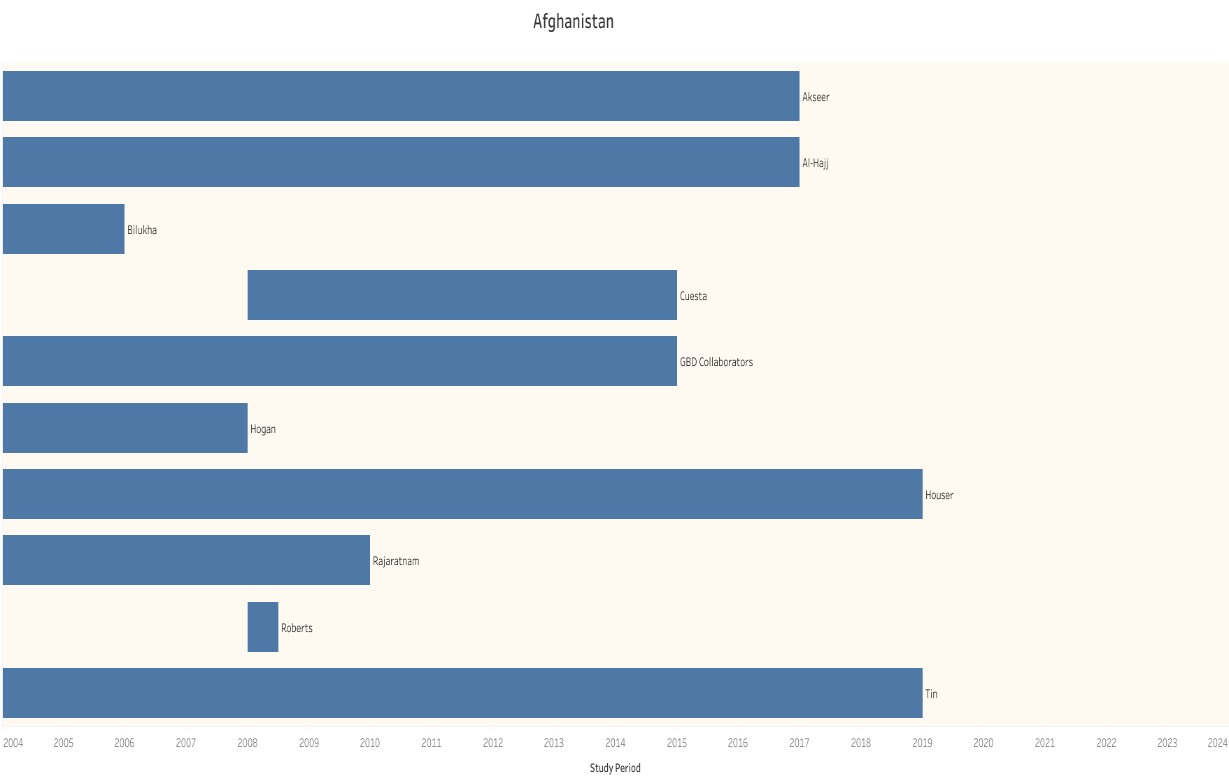


**Figure H: The periods covered by each peer-reviewed study about Afghanistan**


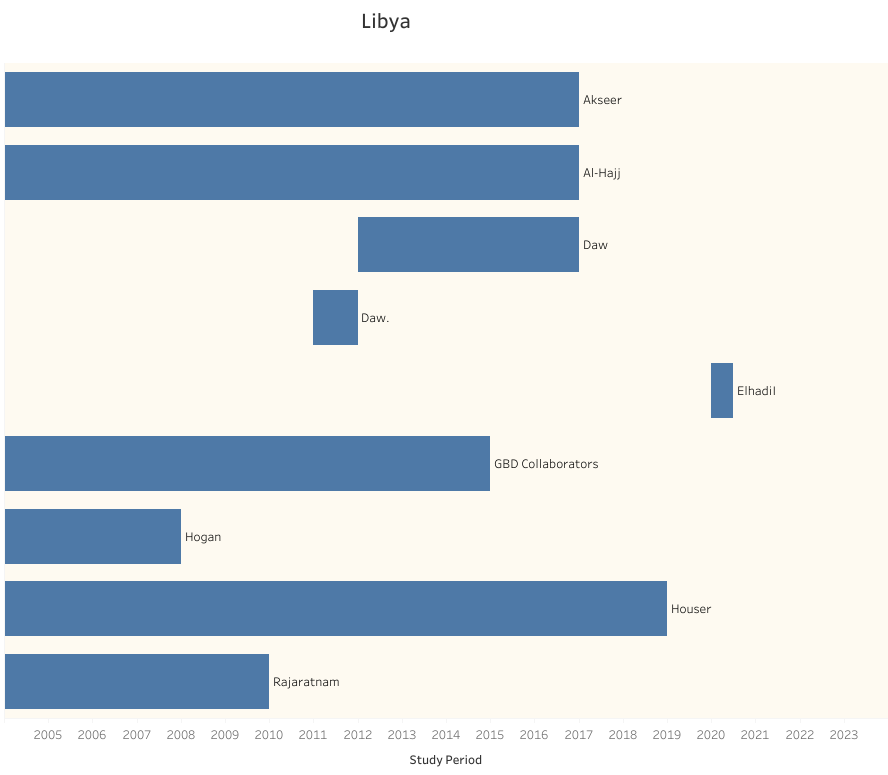


**Figure I: The periods covered by each peer-reviewed study about Libya**


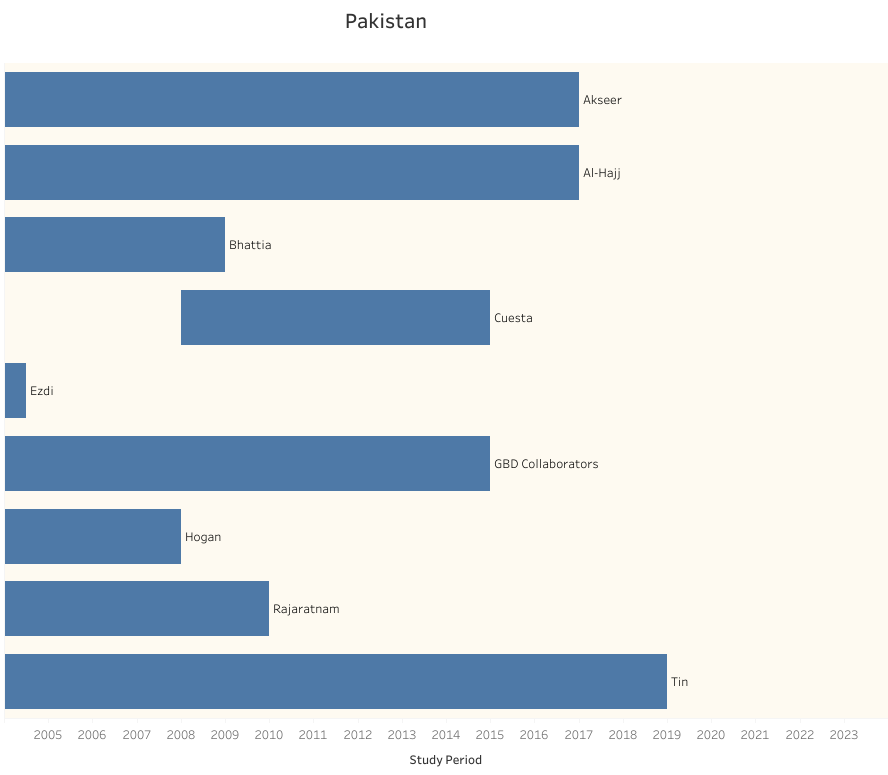


**Figure J: The periods covered by each peer-reviewed study about Pakistan**
