# Supplementary material for: Measuring conflict related mortality in ten countries of the WHO Eastern Mediterranean Region (2004–2024): A scoping review
Source: PLOS Glob Public Health. 2025 Nov 11;5(11):e0005465. doi: 10.1371/journal.pgph.0005465 (PMC12604791; doi:10.1371/journal.pgph.0005465)
Supplement: S4 Text — (DOCX) [file pgph.0005465.s005.docx]

**S4 Text: Details about the conflict-related deaths databases**

**Table A. The list of the included conflict-related deaths databases**

| **The database** | **Affiliation** | **Country of affiliation** | **Fund** | **Studied countries** |
| --- | --- | --- | --- | --- |
| Afghanistan Independent Human Rights Commission | A national human rights institution | Afghanistan | The country’s income | Afghanistan |
| Civilian Impact Monitoring Project | United Nations Protection Cluster for Yemen | Not clear | United Nations Protection Cluster for Yemen | Yemen |
| Insight | Independent non-profit organization | Syria | Not clearly mentioned | Syria |
| The South Asia Terrorism Portal | The Institute for Conflict Management | India | Grants and financial assistance from agencies involved in the study of peace and conflict worldwide / individual donations | Afghanistan Pakistan |
| Syrian Centre of Statistics and Research | Independent research centre | Germany | Not clearly mentioned | Syria |
| Syrian Network for Human Rights | Non-governmental, non-profit, independent organization | USA, France, Turkey | Individual donation | Syria |
| B'Tselem | Independent, non-partisan organization | Israeli | Individual donors/ Bread for the World/ Catholic Relief Services/ Christian/ Aid Ireland / Church of Scotland/ Common Services Corporation of the United Church of Christ/ Consulat General de France a Jerusalem/ DanChurchAid/ European Commission/ European Endowment for Democracy/ Euro-Mediterranean Foundation of Support to Human Rights Defenders/ Fondation Pro Victimis/ Human Rights and International Humanitarian Law Secretariat/ Moriah Fund/ New Israel Fund/ Office for the Coordination of Humanitarian Affairs/ Open Society Foundations/ Royal Norwegian Embassy/ Sigrid Rausing Trust/ Swedish International Development Cooperation Agency/ Stichting Niks Voor Niks/ Trocaire/ United Nations Development Programme/ United Nations Children's Fund/ United Nations Entity for Gender Equality and the Empowerment of Women | Palestine |
| Global Terrorism Database | National Consortium for the Study of Terrorism and Responses to Terrorism, University of Maryland | USA | United States National Institute of Justice/ United States Department of Homeland Security, Science and Technology Directorate/ United States Department of State, Bureau of Counterterrorism and Countering Violent Extremism/ United States Department of Defense, Combating Terrorism Technical Support Office/ German Federal Foreign Office/ United Kingdom Foreign, Commonwealth, and Development Office/ United States Department of Defense, Basic Research Office | All |
| The International Crisis Behaviour | Duke University and USC University of South California | USA | Not clearly mentioned | All |
| Integrated Network for Societal Conflict Research | The Center for Systemic Peace | USA | Not clearly mentioned | All |
| Iraq Body Count | Conflict Casualties Monitor, a company | UK | ifa (Institut für Auslandsbeziehungen) with the means of the German Federal Foreign Office / The Joseph Rowntree Charitable Trust / The Funding Network / The Network for Social Change / The Sigrid Rausing Trust | Iraq |
| The RAND Database of Worldwide Terrorism Incidents | Nonprofit research organization | USA | U.S. government agencies/ U.S. state and local governments/ non-U.S. governments, agencies, and ministries/ International organizations / Colleges and universities/ Foundations /Professional associations / Other nonprofit organizations / Industry Philanthropic support | All |
| Yemen Data Project | Not-for-profit organization | Not clear | Open Society Foundations/ Joseph Rowntree Charitable Trust/ Pro bono contributions from volunteers | Yemen |
| The Peace Research Institute Oslo | Independent foundation | Norway | Basic core grant from the Research Council of Norway / Various national and international sources. | All |
| Uppsala Conflict Data Program | Department of Peace and Conflict Research at Uppsala University | Sweden | Riksbankens Jubileumsfond (RJ)/ Uppsala universitet/ Swedish Research Council | All |
| Airwars | Not-for-profit company | UK | Joseph Rowntree Charitable Trust / Open Society Foundations / Stichting Democratie en Media/ Reva and David Logan Foundation / J. Leon Foundation / Philanthropic organizations/ Public donations/ Pro bono contributions from volunteers | Iraq, Syria, Libya, Somalia, Palestine |
| Action on Armed Violence | Charity | UK | Not clearly mentioned | All |
| The Armed Conflict Location & Event Data Project | Non-profit organization incorporation | USA | Individual donations | All |
| Correlates of War | The Pennsylvania State University | USA | Complex Risk Analytics Fund/ The European Commission/ The Dutch Ministry of Foreign Affairs/ The Tableau Foundation | All |
| Global Health Data Exchange | Institute for Health Metrics and Evaluation (IHME), at the University of Washington | USA | Not clearly mentioned | All |
| Libya Body Count | Not clear | Not clear | Not clearly mentioned | Libya |
| The Violations Documentation Center in Syria (VDC) | Non-profit organisation | Switzerland | The Asfari Foundation/ Schweizerishce Eidgenossenschaft/ International Media Support/ Open Society Foundations / Federal Department of Foreign Affairs FDFA | Syria |
| Palestinian Center for Human Rights | Not-for-profit company | Palestine | NGOs / Charitable foundations/ Selected governments/ Individual donations/ Smaller organizations | Palestine |

**Table B. The sources of information per database**

| **Databases** | **Data Sources** | | | | |
| --- | --- | --- | --- | --- | --- |
|  | **Governmental sources** | **Humanitarian and research organizations** | **Media** | **Primary data collection** | **Other** |
| **Afghanistan Independent Human Rights Commission** | - | - | - | Field monitoring and investigation | - |
| **Civilian Impact Monitoring Project** | - | - | Media and social media | - | - |
| **Insight** | - | Local organizations (not specified) | - | Primary investigations by field correspondents and investigators | Open sources (not specified), activists, and legal experts collected data on individual violations |
| **The South Asia Terrorism Portal** | - | - | Compiled from news reports | - | - |
| **Syrian Centre of Statistics and Research** | - | - | - | Primary research | Secondary resources  (not specified) |
| **Palestinian Center for Human Rights** | - | - | - | Information is gathered by field workers in the Gaza Strip from victims and witnesses of human rights violations. | - |
| **Syrian Network for Human Rights** | - | - | - | First-hand sources, including the victim’s family members and eyewitnesses | Public submissions through a form on the SNHR website, Photos and videos of the victim and incident location, and Internet sources (not specified) |
| **B'Tselem** | Government reports, legal documents | NGO  (not specified) | Local and international news, social media monitoring | Field Research and Testimonies: Firsthand accounts, site visits, and interviews. | Photographic and Video Evidence |
| **Global Terrorism Database** | Court records and government reports | - | Media articles | - | Existing Data Sets, Books, previous publications, Specialized Archives, The Australian Turkish Media Groups report on Armenian Terrorism, Submissions to the Commission for Truth and Reconciliation by the ANC |
| **Databases** | **Governmental sources** | **Humanitarian and research organizations** | **Media** | **Primary data collection** | **Other** |
| **The International Crisis Behaviour** | Governmental reports | NGOs (not specified) | Media | - | Data archives, books |
| **Iraq Body Count** | Official Iraqi sources, such as Medico-Legal Institutes (morgues) and the Ministry of Health | Non-political NGOs, like the Iraqi Red Crescent | News Media | Primary sources such as survivors, eyewitnesses, family members, emergency medics, local police, and various officials | - |
| **Yemen Data Project** | Official records from local authorities | Reports from international and national NGOs, Reports by international human rights groups | Local and international news agencies and media reports, social media accounts (Twitter, Facebook, YouTube, WhatsApp) | - | - |
| **The Peace Research Institute Oslo** | Official death statistics | - | News reports | - | Archival sources |
| **Uppsala Conflict Data Program** | - | Reports and data from NGO and international organizations (like the UN), truth commission reports | News reports, including local and specialized news sources or social media outlets | - | Case studies, historical archives |
| **Airwars** | Military Reporting: Official military statements and reports | Civic Society Groups: International and local organizations | News Outlets: Local and international news reports, social media: Platforms like Twitter, Facebook, and others | - | Non-State Actor Claims: Statements and claims from various non-state actors |
| **Libya Body Count** | Libyan Ministry of Health, Interior Ministry, and Defense Ministry | - | Media reports / social media | - | - |
| **Action on Armed Violence** | - | - | English-language media reports, the ten most used being The New York Times, Xinhua, Reuters, ANI, Anadolu Agency, Al Jazeera, Ukrinform, DAWN, Associated Press, and Myanmar Now | - | - |
| **The Armed Conflict Location & Event Data Project** | - | International institutions and non-governmental organizations | Traditional Media & New Media "social media" (targeted and verified) | - | Local Partner (Local conflict observatories) |
| **The Violations Documentation Center in Syria (VDC)** | - | - | Media centres | Data collectors, a group of human rights and field activists, as well as reporters assigned by the center in different regions | Field hospitals, Cemeteries |
| **Global Health Data Exchange** | **-** | IPUMS, ICPSR, WHO Global Health Observatory, United Nations Population Division, WHO Human Mortality Database, World Bank Open Data, European Health for All Database, CDC Wonder, Health Indicators Warehouse, International institute of strategic studies, Amnesty | - | - | International Household Survey Network, Online Data Archive for Population Studies (SodaPop), Synapse (formerly Sage Bionetworks Repository), HealthData.Gov, Inter-University Consortium for Political and Social Research, 2.Dataverse from the Institute for Quantitative Social Science at Harvard University, Data Sharing Sites (Open Microdata, AidData, Humanitarian Data Exchange, Other Cool Data Sites, Wolfram Alpha) |
| **Correlates of War** | Not clearly described | | | | |
| **Integrated Network for Societal Conflict Research** |  |  |  |  |  |
| **The RAND Database of Worldwide Terrorism Incidents** |  |  |  |  |  |

**Table C. The methods used by the databases reporting conflict-related deaths**

| **Methods** | | **N** | **%** |
| --- | --- | --- | --- |
| Type of data | Primary | 2 | 9 |
|  | Secondary | 11 | 48 |
|  | Mixed primary and secondary | 7 | 30 |
|  | Not clear | 3 | 13 |
| The source of information | Governmental reports or websites | 8 | 35 |
|  | Humanitarian and research organizations | 9 | 39 |
|  | Media | 14 | 61 |
|  | Primary data collection | 8 | 35 |
|  | Other | 14 | 61 |
| Data stratification | Age | 13 | 57 |
|  | Sex | 12 | 52 |
|  | The type of weapon used | 8 | 35 |
|  | Cause of death | 2 | 9 |
|  | Geographical location | 21 | 91 |
|  | Alleged perpetrator | 20 | 87 |
| Reported outcomes | Total number of deaths | 23 | 100 |
|  | Crude mortality rate | 1 | 4 |
|  | Conflict and terrorism proportionate mortality | 1 | 4 |
|  | Total number of injuries | 10 | 43 |
|  | YLDs and DALYs | 1 | 4 |
