# Supplementary material for: Measuring conflict related mortality in ten countries of the WHO Eastern Mediterranean Region (2004–2024): A scoping review
Source: PLOS Glob Public Health. 2025 Nov 11;5(11):e0005465. doi: 10.1371/journal.pgph.0005465 (PMC12604791; doi:10.1371/journal.pgph.0005465)
Supplement: S5 Text — (DOCX) [file pgph.0005465.s006.docx]

**S5 Text: Details about the nutrition and mortality surveys**

**Table A. Characteristics of nutrition and mortality surveys (N=114)**

|  | | **n** | **%** |
| --- | --- | --- | --- |
| **Country** | Yemen | 51 | 45 |
|  | Sudan | 26 | 23 |
|  | Pakistan | 13 | 11 |
|  | Somalia | 12 | 11 |
|  | Afghanistan | 10 | 9 |
|  | Syria | 1 | 1 |
|  | Iraq | 1 | 1 |
| **Publication date** | 2005 | 2 | 2 |
|  | 2012 | 11 | 10 |
|  | 2013 | 4 | 4 |
|  | 2014 | 11 | 10 |
|  | 2015 | 5 | 4 |
|  | 2016 | 8 | 7 |
|  | 2017 | 4 | 4 |
|  | 2018 | 11 | 10 |
|  | 2019 | 4 | 4 |
|  | 2020 | 2 | 2 |
|  | 2021 | 2 | 2 |
|  | 2022 | 13 | 11 |
|  | 2023 | 22 | 19 |
|  | 2024 | 14 | 12 |
|  | Missing | 1 | 1 |
| **Partner organizations/ Institutions involved in the survey (other than local health authorities)** | UNICEF | 71 | 62 |
|  | Action Against Hunger | 43 | 38 |
|  | Save the Children | 16 | 14 |
|  | WHO | 10 | 9 |
|  | Contech International | 7 | 6 |
|  | WFP | 7 | 6 |
|  | IRC | 4 | 4 |
|  | Caafimaad | 3 | 3 |
|  | IOM | 3 | 3 |
|  | European Commission Humanitarian Aid and Civil Protection | 2 | 2 |
|  | Other | 32 | 28 |
| **Organizations/ Institutions funded the surveys** | UNICEF | 58 | 51 |
|  | European Civil Protection and Humanitarian Aid Operations | 34 | 30 |
|  | Global Affairs Canada | 9 | 8 |
|  | Save the Children | 9 | 8 |
|  | Swedish International Development Cooperation Agency | 6 | 5 |
|  | USAID | 5 | 4 |
|  | World Food Programme | 5 | 4 |
|  | UK Department for International Development | 4 | 4 |
|  | OCHA | 4 | 4 |
|  | WHO | 2 | 2 |
|  | IRC | 2 | 2 |
|  | IOM | 2 | 2 |
|  | Not clear | 12 | 11 |
|  | Other | 17 | 15 |

^a Note: percentages were rounded to the nearest full digit, as result percentages may not always perfectly add up to 100%.^

**Table B. Methods used, reported outcomes, and challenges of the nutrition and mortality surveys (N=114)**

|  | | **n** | **%** |
| --- | --- | --- | --- |
| **Scope** | Sub-national | 113 | 99 |
|  | National | 1 | 1 |
| **Type of the survey** | SMART survey | 113 | 99 |
|  | Not SMART survey | 1 | 1 |
| **Targeted population** | All population | 110 | 97 |
|  | Internally Displaced Persons | 4 | 4 |
| **Data verification methods** | Confirmed by the supervisor | 31 | 27 |
|  | Verification by field revisit "if needed" | 12 | 11 |
|  | Not mentioned | 71 | 62 |
| **Reported outcomes** | CMR | 112 | 98 |
|  | Age-specific mortality | 110 | 97 |
| **Stratification of data** | Age | 108 | 95 |
|  | Sex | 56 | 49 |
|  | Geographical Location | 39 | 34 |
|  | Cause of Death | 36 | 32 |
| **Challenges in Obtaining Data** | Reported challenges | 47 | 41 |
|  | Didn’t report challenges | 67 | 59 |
| **Challenges (N=47)** | Inaccessibility due to security concerns/ loss of data due to a security issue | 34 | 72 |
|  | Geographical inaccessibility | 9 | 19 |
|  | Recall bias | 8 | 17 |
|  | Cultural factors that hinder the data collection ^b^ | 8 | 17 |
|  | Logistics (Lack of or poor connectivity) | 1 | 2 |

^a Note: percentages were rounded to the nearest full digit, as result percentages may not always perfectly add up to 100%.^

^b the cultural factors including: reluctance to share information, sensitivity around mortality data (especially the cause of death), and cultural restrictions on female data collectors.^
